# Supplementary material for: HIVprotI: an integrated web based platform for prediction and design of HIV proteins inhibitors
Source: J Cheminform. 2018 Mar 9;10:12. doi: 10.1186/s13321-018-0266-y (PMC5845081; doi:10.1186/s13321-018-0266-y)
Supplement: Supplementary file 2 — Additional file 2. Source code of HIVProtI web server. [file 13321_2018_266_MOESM2_ESM.zip › HIVprotI_Source-code/hivprotI/weka-3-6-6/doc/weka/core/converters/DatabaseSaver.html]

DatabaseSaver


---


|  |  |  |
| --- | --- | --- |
| **Overview** \| **Package** \| **Class** \| **Tree** \| **Deprecated** \| **Index** \| **Help** \| **Weka's home** | |  |
| **PREV CLASS**   **NEXT CLASS** | **FRAMES**    **NO FRAMES**     **All Classes** |  |
| SUMMARY: NESTED \| FIELD \| CONSTR \| METHOD | DETAIL: FIELD \| CONSTR \| METHOD |  |


---


## weka.core.converters Class DatabaseSaver

```
java.lang.Object
  weka.core.converters.AbstractSaver
      weka.core.converters.DatabaseSaver
```

**All Implemented Interfaces:**: java.io.Serializable, CapabilitiesHandler, BatchConverter, DatabaseConverter, IncrementalConverter, Saver, OptionHandler, RevisionHandler

---

``` public class DatabaseSaver extends AbstractSaver implements BatchConverter, IncrementalConverter, DatabaseConverter, OptionHandler ```

Writes to a database (tested with MySQL, InstantDB, HSQLDB).


Valid options are:

```
 -url <JDBC URL>
  The JDBC URL to connect to.
  (default: from DatabaseUtils.props file)
```

```
 -user <name>
  The user to connect with to the database.
  (default: none)
```

```
 -password <password>
  The password to connect with to the database.
  (default: none)
```

```
 -T <table name>
  The name of the table.
  (default: the relation name)
```

```
 -P
  Add an ID column as primary key. The name is specified
  in the DatabaseUtils file ('idColumn'). The DatabaseLoader
  won't load this column.
```

```
 -i <input file name>
  Input file in arff format that should be saved in database.
```

**Version:**
:   $Revision: 7499 $

**Author:**
:   Stefan Mutter (mutter@cs.waikato.ac.nz)

**See Also:**: Serialized Form

---

**Field Summary**

**Fields inherited from interface weka.core.converters.Saver**

`BATCH, INCREMENTAL, NONE`


**Constructor Summary**

`DatabaseSaver()`
  
          Constructor.


| **Method Summary** | |
| --- | --- |
| `java.lang.String` | `autoKeyGenerationTipText()`             Returns the tip text for this property. |
| `void` | `cancel()`             Cancels the incremental saving process and tries to drop the table if the write mode is CANCEL. |
| `void` | `connectToDatabase()`             Opens a connection to the database. |
| `boolean` | `getAutoKeyGeneration()`             Gets whether or not a primary key will be generated automatically. |
| `Capabilities` | `getCapabilities()`             Returns the Capabilities of this saver. |
| `java.lang.String[]` | `getOptions()`             Gets the setting. |
| `java.lang.String` | `getPassword()`             Returns the database password. |
| `boolean` | `getRelationForTableName()`             Gets whether or not the relation name is used as name of the table. |
| `java.lang.String` | `getRevision()`             Returns the revision string. |
| `java.lang.String` | `getTableName()`             Gets the table's name. |
| `java.lang.String` | `getUrl()`             Gets the database URL. |
| `java.lang.String` | `getUser()`             Gets the database user. |
| `java.lang.String` | `globalInfo()`             Returns a string describing this Saver. |
| `java.util.Enumeration` | `listOptions()`             Lists the available options. |
| `static void` | `main(java.lang.String[] options)`             Main method. |
| `java.lang.String` | `passwordTipText()`             Returns the tip text for this property. |
| `java.lang.String` | `relationForTableNameTipText()`             Returns the tip text fo this property. |
| `void` | `resetOptions()`             Resets the Saver ready to save a new data set. |
| `void` | `setAutoKeyGeneration(boolean flag)`             En/Dis-ables the automatic generation of a primary key. |
| `void` | `setDestination()`             Sets the database url using the DatabaseUtils file. |
| `void` | `setDestination(java.lang.String url)`             Sets the database url. |
| `void` | `setDestination(java.lang.String url, java.lang.String userName, java.lang.String password)`             Sets the database url. |
| `void` | `setOptions(java.lang.String[] options)`             Sets the options. |
| `void` | `setPassword(java.lang.String password)`             Sets the database password. |
| `void` | `setRelationForTableName(boolean flag)`             En/Dis-ables that the relation name is used for the name of the table (default enabled). |
| `void` | `setTableName(java.lang.String tn)`             Sets the table's name. |
| `void` | `setUrl(java.lang.String url)`             Sets the database URL. |
| `void` | `setUser(java.lang.String user)`             Sets the database user. |
| `java.lang.String` | `tableNameTipText()`             Returns the tip text for this property. |
| `java.lang.String` | `urlTipText()`             Returns the tip text for this property. |
| `java.lang.String` | `userTipText()`             Returns the tip text for this property. |
| `void` | `writeBatch()`             Writes a Batch of instances. |
| `void` | `writeIncremental(Instance inst)`             Saves an instances incrementally. |

**Methods inherited from class weka.core.converters.AbstractSaver**

`filePrefix, getFileExtension, getInstances, getWriteMode, resetStructure, retrieveDir, setDestination, setDestination, setDir, setDirAndPrefix, setFile, setFilePrefix, setInstances, setRetrieval, setStructure`

**Methods inherited from class java.lang.Object**

`equals, getClass, hashCode, notify, notifyAll, toString, wait, wait, wait`

**Constructor Detail**

### DatabaseSaver

```
public DatabaseSaver()
              throws java.lang.Exception
```

:   Constructor.

    **Throws:**: `java.lang.Exception` - throws Exception if property file cannot be read


**Method Detail**

### resetOptions

```
public void resetOptions()
```

:   Resets the Saver ready to save a new data set.

    :   **Overrides:**: `resetOptions` in class `AbstractSaver`

---


### cancel

```
public void cancel()
```

:   Cancels the incremental saving process and tries to drop the table if
    the write mode is CANCEL.

    :   **Overrides:**: `cancel` in class `AbstractSaver`

---


### globalInfo

```
public java.lang.String globalInfo()
```

:   Returns a string describing this Saver.

    :   **Returns:**: a description of the Saver suitable for displaying in the explorer/experimenter gui

---


### setTableName

```
public void setTableName(java.lang.String tn)
```

:   Sets the table's name.

    :   **Parameters:**: `tn` - the name of the table

---


### getTableName

```
public java.lang.String getTableName()
```

:   Gets the table's name.

    :   **Returns:**: the table's name

---


### tableNameTipText

```
public java.lang.String tableNameTipText()
```

:   Returns the tip text for this property.

    :   **Returns:**: the tip text for this property

---


### setAutoKeyGeneration

```
public void setAutoKeyGeneration(boolean flag)
```

:   En/Dis-ables the automatic generation of a primary key.

    :   **Parameters:**: `flag` - flag for automatic key-genereration

---


### getAutoKeyGeneration

```
public boolean getAutoKeyGeneration()
```

:   Gets whether or not a primary key will be generated automatically.

    :   **Returns:**: true if a primary key column will be generated, false otherwise

---


### autoKeyGenerationTipText

```
public java.lang.String autoKeyGenerationTipText()
```

:   Returns the tip text for this property.

    :   **Returns:**: tip text for this property

---


### setRelationForTableName

```
public void setRelationForTableName(boolean flag)
```

:   En/Dis-ables that the relation name is used for the name of the table (default enabled).

    :   **Parameters:**: `flag` - if true the relation name is used as table name

---


### getRelationForTableName

```
public boolean getRelationForTableName()
```

:   Gets whether or not the relation name is used as name of the table.

    :   **Returns:**: true if the relation name is used as the name of the table, false otherwise

---


### relationForTableNameTipText

```
public java.lang.String relationForTableNameTipText()
```

:   Returns the tip text fo this property.

    :   **Returns:**: the tip text for this property

---


### setUrl

```
public void setUrl(java.lang.String url)
```

:   Sets the database URL.

    :   **Specified by:**: `setUrl` in interface `DatabaseConverter`
    :   **Parameters:**: `url` - the URL

---


### getUrl

```
public java.lang.String getUrl()
```

:   Gets the database URL.

    :   **Specified by:**: `getUrl` in interface `DatabaseConverter`
    :   **Returns:**: the URL

---


### urlTipText

```
public java.lang.String urlTipText()
```

:   Returns the tip text for this property.

    :   **Returns:**: the tip text for this property

---


### setUser

```
public void setUser(java.lang.String user)
```

:   Sets the database user.

    :   **Specified by:**: `setUser` in interface `DatabaseConverter`
    :   **Parameters:**: `user` - the user name

---


### getUser

```
public java.lang.String getUser()
```

:   Gets the database user.

    :   **Specified by:**: `getUser` in interface `DatabaseConverter`
    :   **Returns:**: the user name

---


### userTipText

```
public java.lang.String userTipText()
```

:   Returns the tip text for this property.

    :   **Returns:**: the tip text for this property

---


### setPassword

```
public void setPassword(java.lang.String password)
```

:   Sets the database password.

    :   **Specified by:**: `setPassword` in interface `DatabaseConverter`
    :   **Parameters:**: `password` - the password

---


### getPassword

```
public java.lang.String getPassword()
```

:   Returns the database password.

    :   **Returns:**: the database password

---


### passwordTipText

```
public java.lang.String passwordTipText()
```

:   Returns the tip text for this property.

    :   **Returns:**: the tip text for this property

---


### setDestination

```
public void setDestination(java.lang.String url,
                           java.lang.String userName,
                           java.lang.String password)
```

:   Sets the database url.

    :   **Parameters:**: `url` - the database url: `userName` - the user name: `password` - the password

---


### setDestination

```
public void setDestination(java.lang.String url)
```

:   Sets the database url.

    :   **Parameters:**: `url` - the database url

---


### setDestination

```
public void setDestination()
```

:   Sets the database url using the DatabaseUtils file.

---


### getCapabilities

```
public Capabilities getCapabilities()
```

:   Returns the Capabilities of this saver.

    :   **Specified by:**: `getCapabilities` in interface `CapabilitiesHandler` **Overrides:**: `getCapabilities` in class `AbstractSaver`
    :   **Returns:**: the capabilities of this object **See Also:**: `Capabilities`

---


### connectToDatabase

```
public void connectToDatabase()
```

:   Opens a connection to the database.

---


### writeIncremental

```
public void writeIncremental(Instance inst)
                      throws java.io.IOException
```

:   Saves an instances incrementally. Structure has to be set by using the
    setStructure() method or setInstances() method. When a structure is set, a table is created.

    :   **Specified by:**: `writeIncremental` in interface `Saver` **Overrides:**: `writeIncremental` in class `AbstractSaver`
    :   **Parameters:**: `inst` - the instance to save **Throws:**: `java.io.IOException` - throws IOEXception.

---


### writeBatch

```
public void writeBatch()
                throws java.io.IOException
```

:   Writes a Batch of instances.

    :   **Specified by:**: `writeBatch` in interface `Saver` **Specified by:**: `writeBatch` in class `AbstractSaver`
    :   **Throws:**: `java.io.IOException` - throws IOException

---


### getOptions

```
public java.lang.String[] getOptions()
```

:   Gets the setting.

    :   **Specified by:**: `getOptions` in interface `OptionHandler`
    :   **Returns:**: the current setting

---


### listOptions

```
public java.util.Enumeration listOptions()
```

:   Lists the available options.

    :   **Specified by:**: `listOptions` in interface `OptionHandler`
    :   **Returns:**: an enumeration of the available options

---


### setOptions

```
public void setOptions(java.lang.String[] options)
                throws java.lang.Exception
```

:   Sets the options.


    Valid options are:

    ```
     -url <JDBC URL>
      The JDBC URL to connect to.
      (default: from DatabaseUtils.props file)
    ```

    ```
     -user <name>
      The user to connect with to the database.
      (default: none)
    ```

    ```
     -password <password>
      The password to connect with to the database.
      (default: none)
    ```

    ```
     -T <table name>
      The name of the table.
      (default: the relation name)
    ```

    ```
     -P
      Add an ID column as primary key. The name is specified
      in the DatabaseUtils file ('idColumn'). The DatabaseLoader
      won't load this column.
    ```

    ```
     -i <input file name>
      Input file in arff format that should be saved in database.
    ```

    :   **Specified by:**: `setOptions` in interface `OptionHandler`
    :   **Parameters:**: `options` - the options **Throws:**: `java.lang.Exception` - if options cannot be set

---


### getRevision

```
public java.lang.String getRevision()
```

:   Returns the revision string.

    :   **Specified by:**: `getRevision` in interface `RevisionHandler`
    :   **Returns:**: the revision

---


### main

```
public static void main(java.lang.String[] options)
```

:   Main method.

    :   **Parameters:**: `options` - should contain the options of a Saver.


---


|  |  |  |
| --- | --- | --- |
| **Overview** \| **Package** \| **Class** \| **Tree** \| **Deprecated** \| **Index** \| **Help** \| **Weka's home** | |  |
| **PREV CLASS**   **NEXT CLASS** | **FRAMES**    **NO FRAMES**     **All Classes** |  |
| SUMMARY: NESTED \| FIELD \| CONSTR \| METHOD | DETAIL: FIELD \| CONSTR \| METHOD |  |


---
